# A Comprehensive Method for Example-Based Color Transfer with Holistic–Local Balancing and Unit-Wise Riemannian Information Gradient Acceleration

**DOI:** 10.3390/e26110918

**Published:** 2024-10-29

**Authors:** Zeyu Wang, Jialun Zhou, Song Wang, Ning Wang

**Affiliations:** 1 School of Electrical and Information Engineering, Zhengzhou University, Zhengzhou 450001, China; wzy_00000@gs.zzu.edu.cn (Z.W.); ieswang@zzu.edu.cn (S.W.); ienwang@zzu.edu.cn (N.W.); 2 Department of Computer Science, Toronto Metropolitan University, Toronto, ON M5B 2K3, Canada

**Keywords:** color transfer, statistical modeling, GMM, optimization on Riemannian manifold

## Abstract

Color transfer, an essential technique in image editing, has recently received significant attention. However, achieving a balance between holistic color style transfer and local detail refinement remains a challenging task. This paper proposes an innovative color transfer method, named BHL, which stands for Balanced consideration of both Holistic transformation and Local refinement. The BHL method employs a statistical framework to address the challenge of achieving a balance between holistic color transfer and the preservation of fine details during the color transfer process. Holistic color transformation is achieved using optimal transport theory within the generalized Gaussian modeling framework. The local refinement module adjusts color and texture details on a per-pixel basis using a Gaussian Mixture Model (GMM). To address the high computational complexity inherent in complex statistical modeling, a parameter estimation method called the unit-wise Riemannian information gradient (uRIG) method is introduced. The uRIG method significantly reduces the computational burden through the second-order acceleration effect of the Fisher information metric. Comprehensive experiments demonstrate that the BHL method outperforms state-of-the-art techniques in both visual quality and objective evaluation criteria, even under stringent time constraints. Remarkably, the BHL method processes high-resolution images in an average of 4.874 s, achieving the fastest processing time compared to the baselines. The BHL method represents a significant advancement in the field of color transfer, offering a balanced approach that combines holistic transformation and local refinement while maintaining efficiency and high visual quality.

## 1. Introduction

Example-based image color style transfer methods have emerged as powerful tools in computer graphics and image processing. Their significance lies in their ability to transfer the color characteristics from the example image to the source image. This allows for various key applications, such as artistic effects transformation [1], photorealistic image stylization [2], image illuminance adjustment [3,4], and underwater image enhancement [5,6].

Pioneering work in example-based image color style transfer was presented in [7]. The authors achieved the decorrelation of color channels by transforming RGB images into the Lab color space, leveraging simple statistics, such as mean and standard deviation, to linearly map the color characteristics from one image to another. Building on this foundation, the method introduced in [8] retained operations within the RGB space and utilized mean and covariance to account for the inherent correlations between the three color channels. Furthermore, the method detailed in [9] accomplished one-to-one color mapping by transferring the color palette of the example image to the source image through an iterative algorithm that transforms one probability density function into another. In [10], a linear color transformation derived from the Monge–Kantorovich theory was proposed. Following this, Ref. [11] introduced a regularized discrete optimal transport formulation for color transformation, effectively addressing challenges such as mass conservation relaxation and regularization. The method in [12] employed illuminant matching and optimal color palette mapping to achieve color transfer. Moreover, Ref. [13] tackled the limitations of relaxed optimal transport in color transfer by implementing a non-convex regularized optimal transportation method that enforced one-to-one feature matching while minimizing transport dispersion. The authors of [14] introduced a transformation between Multivariate Generalized Gaussian Distributions (MGGDs), consisting of optimal transportation of the second-order statistics and a stochastic-based shape parameter transformation.

While optimal transportation algorithms offer advantages in computational efficiency and ease of use, they have limitations. Notably, the application of a uniform processing method across all pixels hinders the ability to ensure the reasonableness of transformation results in all image regions. This may lead to artifacts, unnatural colorization, irrational luminosity relationships, biased color positioning, and color vignetting in the output image.

To overcome this limitation, an Expectation-Maximization (EM)-based segmentation method for regional color transfer was introduced in [15]. In [16], a soft color segmentation method was presented for color transfer, using a Gaussian Mixture Model (GMM) to capture broad color patterns with soft labels. The method proposed in [17] utilized an improved EM algorithm and a GMM for the automatic selection of appropriate reference colors within target regions. Focusing on local style variations, the method introduced in [18] leveraged Gaussian clustering to capture fine-grained light and color details within images. This method employed novel source-example cluster mapping policies and achieved style transfer through a combination of parametric color transfer and local chromatic adaptation, allowing for seamless image synthesis while preserving spatial and color coherence. A content-based color transfer method introduced in [19] performed high-level scene analysis for semantic region correspondence and utilized a novel optimization framework to achieve color transfer while preserving spatial layout and visual coherence. For applications in cartoon and fabric color transfer, the method presented in [20] improved color transfer vividness and enhanced detail preservation through image segmentation and the incorporation of a total generalized variation regularizer. A representative superpixel-based method for color transfer was presented in [21], utilizing a fast method that employed approximate nearest neighbor matching with enforced diversity and a fusion framework. Lastly, an L2 divergence-based method for color transfer was described in [22], offering flexibility by accommodating color correspondences and ensuring performance despite potential outlier pairs.

Building upon differential geometry concepts, Ref. [23] introduced a method for per-frame color transform interpolation that minimized curvature. In contrast, the method presented in [24] employed iterative probabilistic color mapping with self-learning filtering and multiscale detail manipulation, minimizing the Kullback–Leibler divergence to enhance color fidelity and detail preservation. To improve robustness, the method in [25] leveraged scattered point interpolation with moving least squares and probabilistic modeling in 3D RGB space, enabling robust color transfer across varying conditions. For more compelling results, the method in [26] considered scene illumination and target gamut constraints, utilizing white balancing, illuminant-aware tone mapping, and gamut-based color mapping techniques. Based on the color homography theorem, the method in [27] decomposed color transfer into chromaticity shift and shading adjustment, represented by a global shading curve. Additionally, a 3D color homography model was introduced in [28], approximating the transformation as a combination of a 3D perspective transform and mean intensity mapping. Addressing color transfer in a two-stage process, the method in [29] first prioritized similarities between source image pixel colors and dominant colors during color mapping, followed by an L0 gradient-preserving detail preservation step to refine large gradients at color region boundaries while maintaining small gradients within regions. The method in [30] tackled color transfer estimation with pixel-to-pixel correspondences using a robust feature-based method. This method utilized an optimal inlier maximization algorithm for outlier handling, combined with a novel structure tensor-based feature detector and descriptor, ensuring reliable color distribution matching across images.

Convolutional Neural Networks (CNNs) have proven remarkably adept at capturing the underlying features of images. This proficiency makes them particularly well suited for image style transfer tasks. Their advantage stems from their ability to learn complex image representations, often referred to as deep features. The most common method leverages these deep features to establish correspondences between the source and example images, subsequently implementing the style transfer [31,32,33,34,35,36].

Under this framework, a method for visual attribute transfer between images with different appearances was introduced in [37], focusing on images with similar semantic structures. This method leveraged deep image analogy and extended PatchMatch to guide semantically meaningful transfers. Similarly, the method proposed in [38] was designed for accurate and coherent color transfer in images with similar semantic structures, employing dense correspondences and local linear models. A local colorization method that allowed for customizable results by incorporating different example images was presented in [39]. Beyond CNN-based methods, a self-supervised Generative Adversarial Network (GAN) for High Dynamic Range (HDR) image color transfer was introduced in [40]. A style representation learning method for arbitrary image style transfer using contrastive learning was proposed in [41]. Meanwhile, a multichannel correlation network (MCCNet) for arbitrary video and image style transfer, which ensures temporal consistency and addresses flickering effects, was proposed in [42]. Detailed reviews of different color transfer techniques can be found in [43,44].

While deep learning methods generally achieve superior performance, they have certain limitations. Extensive training datasets and substantial computational resources are required to train these models. Additionally, their performance is hindered when the source image type is not present in the training data. Once trained, these networks may struggle to adapt to different source image sizes.

Existing methods for color transfer each have their own advantages. However, it is difficult to achieve good performance in all aspects, such as texture preservation, color brightness, and time efficiency. To overcome these challenges, this paper makes the following contributions:

1. This paper proposes a method that balances holistic and local costs, named BHL. The BHL method captures the color information of example images more comprehensively at a holistic level while better preserving the texture details of the source images.
2. A customized optimization method is introduced, based on the Riemannian information gradient and called the uRIG method, to address the high computational time associated with parameter estimation for the MGGD and GMM probability models. By leveraging the second-order acceleration effect of the Riemannian information metric (matrix), the uRIG method significantly enhances the time efficiency of the BHL algorithm.
3. In the preprocessing stage, SLIC (Simple Linear Iterative Clustering) is used to sample mini-batches for subsequent iterations. This ensures that the colors of the image will not be too monotonous when refining local areas.
4. Extensive numerical experiments demonstrate that the BHL method achieves a significant advantage in time complexity over existing color transfer techniques while matching or even surpassing the visual quality of existing methods.

## 2. Methodology

This paper aims to achieve fast and high-quality image color transfer by leveraging the complementary strengths of holistic and local methods. To this end, we formulate the engineering problem as a numerical optimization problem, as shown in the following equation:

(1) $$\min\left(C_{h}+C_{\ell }\right),$$

where $C_{h}$ and $C_{\ell }$ represent the costs associated with holistic and local color transformations, respectively. Minimizing both terms in Equation (1) theoretically leads to the optimal color transfer solution.

### 2.1. Holistic Cost

In this work, image pixels are represented as 3-dimensional vectors (i.e., corresponding to the 3 channels in the CIE Lab color space). By statistically modeling all pixel values in the example image, its holistic color features are described by a probability distribution, denoted as Pe2. Similarly, the color features of the source image are represented by a distribution Ps2. The holistic color transfer is accomplished through an optimal transport map between Ps2 and Pe2, as optimal transport guarantees that mapping samples from Ps2 to Pe2 minimizes the color difference (i.e., the transport cost). As a result, the color distribution of the transformed image closely aligns with that of the example image. Mathematically, this problem is expressed as:

(2) $$\min_{T}E_{x∼P_{s}}\left[c\left(x,T(x)\right)\right],$$

where *x* represents a random vector following Ps2, T(x) is the optimal transport mapping to be found, and c(x,T(x)) denotes the transport cost. In this work, the Multivariate Generalized Gaussian Distribution (MGGD) is selected as an appropriate probability distribution to model the source image and example image. The MGGD is a generalization of the multivariate Gaussian distribution; since it inherits the advantages of the Gaussian distribution, it offers a more accurate fit to the real probability density of the dataset due to its adjustable shape parameter. The probability density function of the MGGD is defined as follows [45]:

(3) $$p\left(x|\theta \right)={c|\Sigma |}^{-\frac{1}{2}}g_{\beta }\left(\delta \right)\;\mathrm{with}\;\theta =\left(\mu ,\Sigma ,\beta \right),$$

where the *d*-dimensional vector $\mu \in R^{d}$ is the location (mean) parameter. The d×d-dimensional scatter matrix Σ is symmetric positive definite, and |Σ| denotes the determinant of Σ. The real positive value β is the shape parameter. The coefficient *c* is the normalizing constant

(4) $$c=\beta \pi^{-\frac{d}{2}}2^{-\frac{d}{2\beta }}\Gamma \left(d/2\right)/\Gamma \left(d/2\beta \right),$$

where Γ is the Gamma function. The symbol δ denotes the Mahalanobis distance for simplicity

(5) $$\delta ={\left(x-\mu \right)}^{†}\Sigma^{-1}\left(x-\mu \right),$$

where † denotes the vector or matrix transpose. Then, the real-valued function $g_{\beta }\left(\delta \right)$ is

(6) gβ(δ)=exp−δ2β/2.

To conceptually distinguish between the source image and the example image, denote the random vector corresponding to the source image as x and the random vector corresponding to the example image as e. We define X=x12,⋯,xM2 as a sample set of x, representing the source image (whose color is to be transferred), and $E=\left\{e_{1},\cdots ,e_{N}\right\}$ as a sample set of e, representing the example image. It is assumed that X is distributed according to an MGGD, and the same assumption holds for E. The transformation equation of two MGGDs, i.e., the minimum of Equation (2), is given in [14]. This transformation of MGGDs incorporates the following two key elements:

1. The MK (Monge–Kantorovich) linear transportation associated with the scatter matrices of x and e;
2. A stochastic transformation of the shape parameters of x and e.

Denote $U=\{u_{1},\cdots ,u_{M}\}$ as the image that was transformed by MK mapping, ∀m∈{1,⋯,M}. The expression of MK transportation is

(7a) $$T_{MK}:x_{m}\mapsto u_{m}=\Lambda \left(x_{m}-\mu_{x}\right)+\mu_{e}$$

where

(7b) $$\Lambda =\Sigma_{x}^{-\frac{1}{2}}{\left(\Sigma_{x}^{\frac{1}{2}}\Sigma_{e}\Sigma_{x}^{\frac{1}{2}}\right)}^{\frac{1}{2}}\Sigma_{x}^{-\frac{1}{2}}.$$

Since MK transportation is independent of the shape parameter $\beta_{e}$ of the example image, we need to perform a second transfer operation related to $\beta_{x}$ and $\beta_{e}$. Here, we denote $T_{s}$ as this second transfer operation and $v_{m}=T_{s}\left(u_{m}\right)$, ∀m∈{1,⋯,m}, as the output of this transformation. To incorporate the shape parameter $\beta_{e}$ of the example image into the transportation result v, we first need to eliminate the influence of $\beta_{x}$ on the current result u. Then, we apply the influence of $\beta_{e}$ to u to obtain the output result v with the full parameter influence of e. Therefore, we need to introduce the stochastic representation of u [45]

(8) $$u\overset{d}{=}\mu_{e}+\tau_{x}\Sigma_{e}^{\frac{1}{2}}r.$$

where $\overset{d}{=}$ denotes stochastic equality. The *d*-dimensional random vector r follows a uniform distribution on the unit sphere, and $\tau_{x}$ is a positive random variable (independent of r) satisfying the following condition:

(9) $$\tau_{x}^{2\beta_{x}}∼\Gamma \left(\frac{d}{2\beta_{x}},2\right).$$

According to (8), the expression of $T_{s}$ is obtained as follows:

(10) $$\forall m\in \left\{1,\cdots ,M\right\},\;\;T_{s}:u_{m}\mapsto v_{m}=\frac{\tau_{e}\left(u_{m}-\mu_{e}\right)}{∥\Sigma_{e}^{-\frac{1}{2}}\left(u_{m}-\mu_{e}\right)∥}+\mu_{e},$$

where $\tau_{e}$ is randomly sampled according to (9). By combining and $T_{s}$, we can obtain $T_{h}$:

(11) $$\forall m\in \left\{1,\cdots ,M\right\},\;\;T_{h}:x_{m}\mapsto v_{m}=T_{s}\left(T_{MK}\left(x_{m}\right)\right).$$

Ultimately, this optimal transportation problem is equivalent to an optimization problem: the estimation problem of $\theta_{x}=\left(\mu_{x},\Sigma_{x},\beta_{x}\right)$ and $\theta_{e}=\left(\mu_{e},\Sigma_{e},\beta_{e}\right)$

(12a) $$\min C_{h,x}\left(\theta_{x}\right)\;\;\mathrm{with}\;\;C_{h,x}\left(\theta_{x}\right)=-\frac{1}{M}\sum_{m=1}^{M}\ln p\left(x_{m}|\theta_{x}\right),$$

(12b) $$\min C_{h,e}\left(\theta_{e}\right)\;\;\mathrm{with}\;\;C_{h,e}\left(\theta_{e}\right)=-\frac{1}{N}\sum_{n=1}^{N}\ln p\left(e_{n}|\theta_{e}\right).$$

### 2.2. Local Costs

After the holistic transformation, a local transformation method is expected to adjust the details. In this work, we employ the framework introduced in [46], which achieves state-of-the-art performance. The interest of this framework not only resides in its performance but also in its operational simplicity.

Here, the output of $T_{h}$ is used as the input for the local transformation. The local transformation leverages the assumption that the example image dataset $E=\{e_{1},\cdots ,e_{N}\}$ follows a specific Gaussian Mixture Model (GMM). The means of this GMM are hypothesized to correspond to the source image. In this way, the matching of pixels and the color transformation between the source image and the example image can be performed simultaneously and adaptively during the parameter estimation process of the GMM, without the need for additional segmentation operations.

Denote $Z=\{z_{1},\cdots ,z_{M}\}$ as the means of this GMM. The probability density function of the GMM followed by the random vector $e_{n}$ is defined as follows:

(13) p(en|ω)=∑m=1M1Mp(en|ωm),with ω={ω1,⋯,ωM},ωm=(zm,σm2I),

where $p(e_{n}|\omega_{m})$ denotes the *m*-th Gaussian component of the GMM and *M* represents the total number of components in the mixture. Following [46], a vector $z_{m}\in R^{d}$ is used as the mean (location parameter) of the *m*-th component, and a diagonal covariance matrix σm2I serves as its scatter matrix. Since the covariance involves only one parameter $\sigma_{m}$ to be estimated, we denote $\omega_{m}$ as $\left(z_{m},\sigma_{m}\right)$ in the following sections for simplicity and ignore the identity matrix I. Additionally, all components are assigned equal weights of 1/M in [46]. The local cost is defined as the negative log-likelihood of the GMM:

(14) $$\begin{array}{cc} & \min C_{\ell }\left(\omega \right),\\ & \;\mathrm{with}\;C_{\ell }\left(\omega \right)=-\sum_{n=1}^{N}\ln\left[\frac{1}{M}\sum_{m=1}^{M}p\left(e_{n}|\omega_{m}\right)\right], \end{array}$$

and for each component of the GMM,

(15) $$p\left(e|\omega_{m}\right)=\frac{1}{{\left(2\pi \right)}^{\frac{d}{2}}}\sigma_{m}^{-\frac{d}{2}}\exp\left(-\frac{1}{2}\sigma_{m}^{-1}{∥e-z_{m}∥}^{2}\right).$$

Then, the color transformation is achieved through a GMM estimation process, utilizing $E=\{e_{1},\cdots ,e_{N}\}$ as the sample data and $V=\{v_{1},\cdots ,v_{M}\}$ (output of $T_{h}$) as the initial value for the means $Z=\{z_{1},\cdots ,z_{M}\}$. In the minimization algorithm, we set

(16) $$\left\{z_{1}^{\left(0\right)},\cdots ,z_{M}^{\left(0\right)}\right\}=\left\{v_{1},\cdots ,v_{M}\right\}.$$

While the Expectation-Maximization (EM) algorithm was applied to address this problem in [46], it is no longer suitable in this context. In Section 3, the uRIG method is introduced, specifically tailored to this problem.

## 3. Optimization Algorithm

### 3.1. Main Algorithm

**Figure 1:**
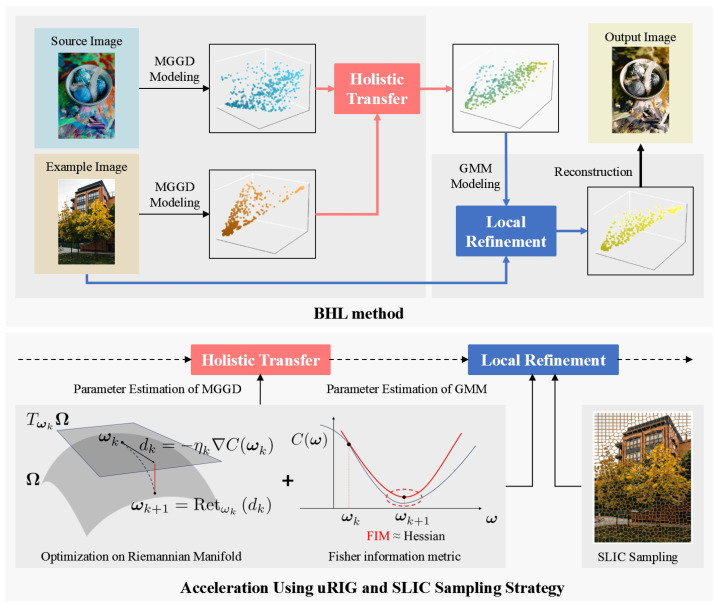
Overview of the BHL method.

To minimize the cost function in Equations (12) and (14), a two-stage optimization process is introduced. Figure 1 illustrates the overall workflow.

During the first stage, we employ Multivariate Generalized Gaussian Distributions (MGGDs) to independently model the source image X={xm2;m=1,⋯,M} and the example image E={en2;n=1,⋯,N}. We then estimate the parameters for these respective models. Utilizing these estimated parameters, we construct the holistic transformation equation Th2 and subsequently apply it to achieve holistic color transfer.

The output of the first stage, denoted as V={vm2;m=1,⋯,M}, serves as the input for the second stage. Here, a GMM with means denoted by Z={zm2;m=1,⋯,M} is leveraged. We posit that the example image E constitutes a sample set for this GMM. Through an iterative maximum likelihood estimation process initialized with Z2(0)=V, the estimated $\hat{Z}$ represents the refined result, which is also the final transformed image. The BHL method is summarized in the Algorithm 1 as below.
**Algorithm 1** The BHL method**Require:** 
Source image $X=\{x_{1},\cdots ,x_{M}\}$, example image $E=\{e_{1},\cdots ,e_{N}\}$; 1:${\hat{\theta }}_{x}$← MGGDESTIMATION(X); 2:${\hat{\theta }}_{e}$← MGGDESTIMATION(E); 3:$\left\{v_{1},\cdots ,v_{M}\right\}$← TRANSMGGD(X, ${\hat{\theta }}_{x}$, ${\hat{\theta }}_{e}$); 4:$\left\{{\hat{z}}_{1},\cdots ,{\hat{z}}_{M}\right\}$← GMMESTIMATION(E, V);**Ensure:** 
Edited image $\hat{Z}=\left\{{\hat{z}}_{1},\cdots ,{\hat{z}}_{M}\right\}$; 5:**procedure** TransMGGD(X, ${\hat{\theta }}_{x}$, ${\hat{\theta }}_{e}$) 6:    Holistic transformation via (11); 7:    **return** $V=\{v_{1},\cdots ,v_{M}\}$; 8:**end procedure**

This color transfer method necessitates solving three optimization problems: (12a), (12b), and (14) (refer to Steps 1 and 2 in Algorithm 1). Traditionally, these problems are addressed using the fixed-point iteration method for (12a) and (12b), and the Expectation-Maximization (EM) algorithm for (14). However, these methods become computationally expensive, particularly for high-resolution images, due to increased time and memory demands. To overcome this computational bottleneck, the unit-wise Riemannian information gradient (uRIG) method is introduced. The core idea of uRIG leverages two key mathematical concepts: the Riemannian manifold and the Fisher information metric [47,48]. Updates on the Riemannian manifold effectively bypass numerical instabilities often encountered with nonlinear constraints, such as positive definite matrices, leading to a more robust algorithm. Additionally, the use of the Fisher information metric (matrix) as a replacement for the Hessian matrix eliminates the need for computationally expensive numerical approximations, thereby accelerating the convergence of the estimation process [49].

### 3.2. Minimization of the Holistic Cost

While the shape parameter of an MGGD is known, such as in the Gaussian (β=1) and Laplace (β=0.5) distributions, its closed-form Fisher information metric exists, as detailed in [50,51]. However, when the shape parameter is unknown, its FIM requires solving a system of partial differential equations, which currently lacks a closed-form solution [52]. Inspired by [53], this paper proposes utilizing a unit-wise Riemannian information metric to address both Problem (12) and the subsequent Problem (14). In Problem (12), the MGGD parameters reside in a product space encompassing $R^{d}$ for the location parameter μ, the set $P_{d}$ of d×d-dimensional symmetric positive definite (SPD) matrices for scatter matrix Σ, and $R_{+}$ for the shape parameter β. The spaces $R^{d}$ and $R_{+}$ are special Riemannian manifolds with zero curvature. The space $P_{d}$ is a matrix (Riemannian) manifold with negative curvature. The product of these three spaces is then a Riemannian manifold [47]. We call a subspace in the product space $R^{d}\times P_{d}\times R_{+}$ a unit since it has a closed-form FIM.

**Definition** **1.** *For the MGGD model, the following spaces are defined: (i) The space $R^{d}$ for the location parameter μ is called a unit; (ii) The space $P_{d}$ for the scatter matrix Σ is called a unit; (iii) The space $R_{+}$ for the shape parameter β is called a unit.*

While the interesting manifold (parameter space) is well defined, the proposition below gives the unit-wise FIM.

**Proposition** **1.** *Denote $\Theta =R^{d}\times P_{d}\times R_{+}$ as the parameter space of an MGGD and $T_{\theta }\Theta =T_{\mu }R^{d}\times T_{\Sigma }P_{d}\times T_{\beta }R_{+}$ as the tangent space of θ∈Θ. The unit-wise FIM of this MGGD is*

(17) $$\langle u_{\theta },v_{\theta }\rangle ={\langle u_{\mu },v_{\mu }\rangle }_{\mu }+{\langle u_{\Sigma },v_{\Sigma }\rangle }_{\Sigma }+{\langle u_{\beta },v_{\beta }\rangle }_{\beta },$$

*with*

(18a)uμ,vμμ=Iμuμ†Σ−1vμ,(18b)uΣ,vΣΣ=IΣ,1trΣ−1uΣΣ−1vΣ+IΣ,2trΣ−1uΣtrΣ−1vΣ,(18c)uβ,vββ=Iβuβvβ,

*where tr is the matrix trace. The vectors $u_{\theta }=\left(u_{\mu },u_{\Sigma },u_{\beta }\right)$ and $v_{\theta }=\left(v_{\mu },v_{\Sigma },v_{\beta }\right)$ are elements in $T_{\theta }\Theta$.*

To simplify the notations, we use the symbols $I_{\mu }$, $I_{\Sigma ,1}$, $I_{\Sigma ,1}$, and $I_{\beta }$ to represent the coefficients in the three inner products above. The following remark gives the values of the three information coefficients.

*Remark*: The three inner products involved in Proposition 1 all have closed-form expressions for the constant coefficients, which can be directly used without any numerical approximation. The information constant with respect to μ is

(19a) $$I_{\mu }=\frac{\left[2(\beta -1)+d\right]\Gamma \left(\frac{d-2}{2\beta }\right)}{2^{\frac{1}{\beta }}d{\left(d-2\right)}^{-1}\Gamma \left(\frac{d}{2\beta }\right)},$$

where *d* is the dimension of the image color vector and Γ(·) is the Gamma function. The information constants with respect to Σ are

(19b) $$I_{\Sigma ,1}=\frac{d+2\beta }{2(d+2)}\;\mathrm{and}\;I_{\Sigma ,2}=\frac{\beta -1}{2(d+2)},$$

The information constant with respect to β is

(19c) $$\begin{array}{cc} I_{\beta }= & \frac{1}{\beta^{2}}\{1+{\left(\frac{d}{2\beta }\right)}^{2}\Psi_{1}\left(\frac{d}{2\beta }\right)\\ & +\frac{d}{\beta }\left[\ln2+\Psi_{0}\left(\frac{d}{2\beta }\right)\right]+\frac{d}{2\beta }[{\left(\ln2\right)}^{2}\\ & +\Psi_{0}\left(1+\frac{d}{2\beta }\right)\left[\ln4+\Psi_{0}\left(1+\frac{d}{2\beta }\right)\right]\\ & +\Psi_{1}\left(1+\frac{d}{2\beta }\right)]\}, \end{array}$$

where $\Psi_{0}$ and $\Psi_{1}$ are the digamma and trigamma functions. The proof of Proposition 1 and its remark can be found in Appendix A. After defining the uFIM on the parameter space Θ, we are able to derive the associated Riemannian gradient based on this metric, i.e., the unit-wise Riemannian information gradient.

**Proposition** **2.** *The uRIG of the holistic cost in Equation (12) is under the following form:*

(20) $$\nabla C_{h}\left(\theta \right)=\left(\begin{array}{c} \nabla_{\mu }C_{h}\left(\theta \right)\\ \nabla_{\Sigma }C_{h}\left(\theta \right)\\ \nabla_{\beta }C_{h}\left(\theta \right) \end{array}\right),$$

*where the components of the three parameters are as follows:*

(21a)∇μCh(θ)=−1L∑i=1LβIμ−1δiβ−1(xi−μ),(21b)∇ΣCh(θ)=−1L∑i=1L[βδiβ−12IΣ,1(xi−μ)(xi−μ)†−IΣ,1+IΣ,2βδiβ+12IΣ,1(IΣ,1+dIΣ,2)Σ],(21c)∇βCh(θ)=1L∑i=1L1Iβ{1β1+d2βΨ0d2β+ln2−12βδiβ−1}.

The values of the coefficients $I_{\mu }$, $I_{\Sigma }$, and $I_{\beta }$ are presented in (19). We recall that the symbol $\delta_{i}$ denotes the Mahalanobis distance, defined as $\delta_{i}={\left(x_{i}-\mu \right)}^{†}\Sigma^{-1}\left(x_{i}-\mu \right)$. The symbol $\Psi_{0}$ denotes the digamma function. The proof of Proposition 2 can be found in Appendix B.

Having established the relevant metric and gradient on the manifold, we now turn to the retraction map, which plays an important role in optimization algorithms. The retraction map serves as a bridge between the tangent space and the manifold itself, enabling us to efficiently perform gradient descent.

In Euclidean space, no special treatment is usually required for gradient descent. However, on a manifold, after moving in the direction of descent (i.e., typically the gradient) in the tangent space, a ‘retraction’ operation is needed to ensure that the parameters always remain within the constrained space. Therefore, the ideal retraction map is the geodesic map that performs the ‘retraction’ operation along geodesics. In particular, the geodesic map for Euclidean space is simply vector addition. Then, for the geodesic map on $P_{d}$, we employ the form introduced in [54] in this work. Since each of the three units possesses its own intrinsic geodesic map, the most natural retraction map on Θ is the product of the three geodesic maps.

**Proposition** **3.** *The following map is a retraction on $\Theta =R^{d}\times P_{d}\times R_{+}$:*

(22) $$\begin{array}{cccc} {\mathrm{Ret}}_{\theta }: & T_{\theta }\Theta & \to & \Theta \\ & \left(\begin{array}{c} u_{\mu }\\ u_{\Sigma }\\ u_{\beta } \end{array}\right) & \mapsto & \left(\begin{array}{c} \mu +u_{\mu }\\ \Sigma \mathrm{Exp}\left(\Sigma^{-1}u_{\Sigma }\right)\\ \beta \exp\left(u_{\beta }/\beta \right) \end{array}\right), \end{array}$$

*where $T_{\theta }\Theta =T_{\mu }R^{d}\times T_{\Sigma }P_{d}\times T_{\beta }R_{+}$ is the tangent space at the point θ=(μ,Σ,β), and the vector $\left(u_{\mu },u_{\Sigma },u_{\beta }\right)$ is an element of $T_{\theta }\Theta$.*

In Proposition 3, exp denotes the natural exponential function on the real number field, while Exp refers to the matrix exponential map. The proof of Proposition 3 is presented in Appendix C.

With the foundation of necessary components in place, we now turn our attention to the specific iterative method employed for the estimation of the MGGD.

In the Algorithm 2, ${\mathrm{Ret}}_{\theta }$ is given in (22). Indeed, in practical applications, in pursuit of time efficiency, the iteration is carried out in the form of mini-batch stochastic gradient descent, i.e., the constant *L* in (21) is the mini-batch size. Its convergence analysis and the selection of the coefficient *a* are discussed in Section 3.4, along with the optimization of $C_{\ell }$.
**Algorithm 2** MGGD estimation using the uRIG method**Require:** 
Dataset $X=\{x_{1},\cdots ,x_{M}\}$;**Ensure:** 
estimate ${\hat{\theta }}_{x}$; 1:**for** k=1, 2, 3, ⋯ **do** 2:    Compute uRIG via (20); 3:    Define learning rate $\eta_{k}=a/k;$ 4:    Update $\theta^{\left(k+1\right)}\leftarrow {\mathrm{Ret}}_{\theta^{\left(k\right)}}\left(-\eta_{k}\nabla C_{h}\left(\theta^{\left(k\right)}\right)\right)$ 5:**end for**

### 3.3. Minimization of the Local Cost

We assumed in Section 2.2 that all the component weights of the GMM are equal to 1/M, where M is the number of components. So, the EM algorithm is no longer the most suitable choice when the component weights are pre-set.

Instead, we adopt the uRIG method in the stochastic gradient descent framework. Specifically, for any component, the GMM, $p(e|\omega_{m})$ is a Gaussian distribution, which is a special case of the MGGD with the shape parameter β=1. Therefore, we can directly apply the uRIG of the MGGD to the estimation of the GMM.

Due to the complex parameters of the GMM, we declare the following symbols for the sake of convenience in expression and understanding:

- Parameter: $\omega =\prod_{m=1}^{M}\omega_{m}$, with $\omega_{m}=\left(z_{m},\sigma_{m}\right)$;
- Parameter space: $\Omega =\prod_{m=1}^{M}\Omega_{m}$, with $\Omega_{m}=R^{d}\times R_{+}$;
- Tangent space at point ω: $T_{\omega }\Omega =\prod_{m=1}^{M}T_{\omega_{m}}\Omega_{m}$, with $T_{\omega_{m}}\Omega_{m}=R^{d}\times R$.

Similar to the case of the MGGD, we start with the definition of a unit.

**Definition** **2.** *For any component $p(e|z_{m},\sigma_{m})$ of the GMM defined in Equation (13), we define the following spaces:*

- *The space $R^{d}$ for the location parameter $z_{m}$ is a unit;*
- *The space $R_{+}$ for the covariance $\sigma_{m}$ is a unit.*

In fact, each unit of the GMM parameter space is simply a Euclidean space; therefore, its uFIM is easier to derive. The uFIM is able to be obtained in the following form by taking the second differential of the log-likelihood with respect to each unit.

**Proposition** **4.** *The unit-wise Fisher information metric of the GMM is*

(23) uω,vωω=∑m=1M1σmuzm⊤vzm2+d2σm2 uσmvσm.

*The vectors $u_{\omega }$ and $v_{\omega }$ are elements in $T_{\omega }\Omega$, and $u_{z_{m}}$ and $v_{z_{m}}$ are elements in $T_{\omega_{m}}\Omega_{m}$.*

The proof can be found in Appendix A. Then, the unit-wise information gradient can be easily derived based on the inner product in Equation (23).

**Proposition** **5.** *The unit-wise information gradient of the GMM is*

(24) $$\begin{array}{cc} \nabla C_{\ell }\left(\omega \right)= & (\nabla_{z_{1}}C_{\ell }\left(\omega_{1}\right),\nabla_{\sigma_{1}}C_{\ell }\left(\omega_{1}\right),\cdots ,\\ & \nabla_{z_{m}}C_{\ell }\left(\omega_{m}\right),\nabla_{\sigma_{m}}C_{\ell }\left(\omega_{m}\right),\cdots ,\\ & \nabla_{z_{M}}C_{\ell }\left(\omega_{M}\right),\nabla_{\sigma_{M}}C_{\ell }\left(\omega_{M}\right){)}^{†}, \end{array}$$

*where*

(25a) $$\nabla_{z_{m}}C_{\ell }\left(\omega_{m}\right)=-\frac{1}{N}\sum_{n=1}^{N}o_{m,n}\left(e_{n}-z_{m}\right),$$

(25b) $$\nabla_{\sigma_{m}}C_{\ell }\left(\omega_{m}\right)=\frac{1}{N}\sum_{n=1}^{N}o_{m,n}\left(\sigma_{m}-\frac{∥e_{n}-z_{m}{∥}^{2}}{d}\right),$$

*where $o_{m,n}$ represents the posterior probabilities*

$$o_{m,n}=\frac{p(e_{n}|\omega_{m})}{\sum_{i=1}^{M}p\left(e_{i}|\omega_{i}\right)}.$$

The proof of Proposition 5 is presented in Appendix B. For the means $z_{m}$, the retraction map is vector addition in the Euclidean space $R^{d}$. For the coefficient $\sigma_{m}$ of the covariance, we follow the same treatment as for the shape parameter β in (22). Using their product, the retraction shown below can be obtained.

**Proposition** **6.** *The following map is a retraction on the parameter space of the GMM:*

(26) Retω:TωΩ→Ωuz1uσ1⋮uzmuσm⋮uzMuσM↦z12+uz12σ12exp(σ1−1uσ1)2⋮zm+uzmσm2exp(σm−1uσm)2⋮zM+uzMσM2exp(σM−1uσM)2.

The Algorithm 3 below gives the update rule for parameter estimation of the GMM.
**Algorithm 3** GMM estimation using the uRIG method**Require:** 
Dataset $E=\{e_{1},\cdots ,e_{N}\}$;**Ensure:** 
estimate $\hat{\omega }$; 1:**for** k=1, 2, 3, ⋯ **do** 2:    Compute uRIG via (24); 3:    Define learning rate $\eta_{k}=a/k;$ 4:    Update $\omega^{\left(k+1\right)}\leftarrow {\mathrm{Ret}}_{\omega^{\left(k\right)}}\left(-\eta_{k}\nabla C_{\ell }\left(\omega^{\left(k\right)}\right)\right)$ 5:**end for**

In the above algorithm, $\nabla C_{\ell }\left(\omega^{\left(k\right)}\right)$ and Retω2 are given in (24) and (26), respectively. For the GMM, the uRIG method also performs under the form of mini-batch stochastic gradient descent. The convergence analysis and selection of the coefficient *a* of Algorithms 2 and 3 are discussed in the following subsection.

### 3.4. Convergence Analysis

To simplify the presentation and enhance the clarity of the convergence analysis of Algorithms 2 and 3, we unify the notations in this subsection. In the following part of this subsection, we use ω to represent the parameters and Ω to denote the parameter space (regardless of whether it is MGGD or GMM). Consider a statistical model with *M* units, and let *N* be the number of observed samples.

Since the conditions (1–3) shown below hold, the high time efficiency and robustness of Algorithm 1 can be established, as shown in Proposition 7.

The conditions are as follows:

1. The cost C(ω) has an isolated stationary point at $\omega =\omega^{*}$, where $\omega^{*}$ is the true parameter;
2. There exists a compact and convex neighborhood U⊂Ω of $\omega^{*}$ such that the sequence generated by Algorithm 1 remains within U.
3. The learning rate $\eta_{k}=a/k$, a>0, verifies the usual condition for stochastic approximation:
  ∑k=1+∞ηk2=+∞, ∑k=1∞ηk2<+∞.

**Proposition** **7.** *With these three conditions, we have*

(27) limk→∞ω2(k)=ω*.

The proof of Proposition 7 is shown in Appendix D.

## 4. Experiment

In this section, the BHL method is evaluated from two perspectives: visual assessment and objective quantitative analysis. The BHL method is benchmarked against five state-of-the-art methods known for their high performance. In terms of holistic transformation methods, we chose the representative MGGD transformation method in [14]. In the domain of local transformation methods, we chose two top-performing methods: the GMM-based transformation technique [46] and the method derived from L2 divergence [22]. Two state-of-the-art deep neural network-based methods, specifically the CAST method [41] and MCCNet [42], were also included in the comparative experiments for evaluation.

We randomly selected five groups of images for experimental comparison, i.e., 10 different images, with 5 as the source images and 5 as the example images. All experiments were conducted on a regular laptop with an AMD Ryzen 7 6800H processor and a main frequency of 3.2 GHz.

### 4.1. Parameter Setting

For the uRIG algorithm, the initialization θ2(0) of Algorithm 2 was determined using the method of moments [55], while the initialization of Algorithm 3 was obtained from the output of holistic transformation V, i.e., Z2(0)=V. The coefficient *a* of the learning rate was estimated according to Proposition 2 in [56].

To facilitate gradient descent, a preprocessing step was introduced in [46] to select the mini-batch. Prior to commencing the iterations, for each pixel in Z2(0), *b* nearest neighbors are chosen from E. Notably, *b* corresponds to the size of the mini-batch, and these selected *b* pixels from E then comprise the mini-batch employed for gradient descent. While this method effectively captures color information, it results in a significant computational burden in the preprocessing stage due to the need to sort M×N pixels.

To address this limitation, we proposed a distinct mini-batch sampling strategy that leverages the Simple Linear Iterative Clustering (SLIC) algorithm. Initially, we applied the SLIC algorithm for superpixel segmentation on the example image E, partitioning it into 1000 superpixels. For each pixel in Z2(0), we computed the distance to the mean of each superpixel in E. Based on these distances, the $b^{\prime }$ nearest superpixels were selected to replace the first *b* pixels during the iteration (typically, $b^{\prime }⩽b$). This method offers two main advantages. First, it significantly reduces the sorting time, particularly for high-resolution images. Second, by utilizing all pixels within the superpixels, the hyperparameter $b^{\prime }$ becomes less sensitive to the final output. Specifically, extreme values of $b^{\prime }$ mainly affect the algorithm’s runtime rather than the color richness of the output. Therefore, it is possible to choose a relatively smaller $b^{\prime }$ to reduce time costs. In the experiments, we set b=100 and $b^{\prime }=50$.

This method substantially reduced the preprocessing time complexity, as detailed in Section 4.4. In addition, to incorporate spatial information, the Laplacian regularization term introduced in [46,57] was also applied to Equation (14).

### 4.2. Quantitative Comparison

In existing research [12,18,25,46,58], the Structural Similarity Index Measure (SSIM) and Peak Signal-to-Noise Ratio (PSNR) are commonly employed to assess the textural similarity between the output image V and the source image X. Specifically, the SSIM quantifies the level of artifacts introduced by the color transfer method, while the PSNR measures the mean squared error between the two images. The color style similarity between the output image and the example image is typically evaluated using the Frechet Inception Distance (FID) and Perceptual Hash Value (PHV) [59,60].

The five methods involved in the comparative experiment have their own advantages due to their different processing techniques and optimization goals. However, no method performed well across all four quantitative evaluation criteria at the same time. For example, MCCNet achieved superior structural fidelity visually because it enhanced the edges of objects in the image (such as the edges of petals). However, excessive enhancement led to large artifacts. The L2 method provided bright-colored visual results, but because it requires local matching of the color palette, its output sometimes contained local color deviations. The results of the MGGD method and the BHL method show that their PSNR values were not relatively high. This is because the processing of these two methods requires a resampling operation combined with the parameters of the example image. Noise that differs from the source image will result in a low PSNR value [58].

To evaluate the performance of these methods, we introduced a comprehensive evaluation technique: the Technique for Order Preference by Similarity to Ideal Solution (TOPSIS) [61]. TOPSIS is a multi-criteria decision analysis technique that assesses the performance of candidate methods by calculating their distances to the ideal and negative-ideal solutions, thereby providing a comprehensive assessment.

Quantitative comparisons between the BHL method and five other methods are presented in Table 1, Table 2, Table 3, Table 4 and Table 5. When calculating the TOPSIS score, we performed the necessary order adjustments and normalization steps. The final TOPSIS scores range from 0 to 1, with higher values indicating better overall performance. As shown in Table 1, Table 2, Table 3, Table 4 and Table 5, the BHL method, which balances holistic and local information, achieved the highest TOPSIS scores. Due to its modeling and optimization techniques, the GMM algorithm achieved the best results in terms of the SSIM and PSNR. However, the performance of this algorithm varied with different hyperparameter settings, resulting in differing color effects. Consequently, the GMM method did not perform very well in the FID and PHV criteria. In contrast, the two deep learning methods, CAST and MCCNet, benefited from the robust color feature-capture capabilities of deep neural networks, achieving higher FID and PHV scores. However, these methods fell short in obtaining high textural similarity scores. The L2 divergence-based method showed severe distortions and artifacts in some images, leading to a low PHV score and, consequently, a lower comprehensive evaluation score. The MGGD method, which is based on optimal transport, performed well across all criteria. However, its lack of attention to detail resulted in numerous local artifacts, adversely affecting its evaluation score. Detailed visual comparisons are provided in the next subsection.

### 4.3. Visual Comparison

**Figure 2:**
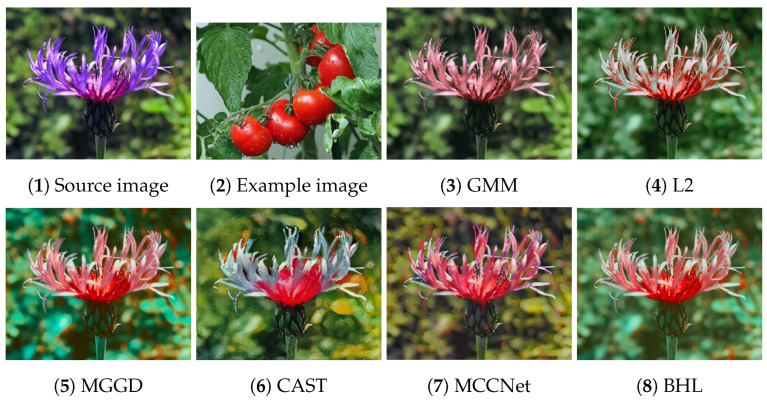
Source image: purple flower; Example image: tomato.

**Figure 3:**
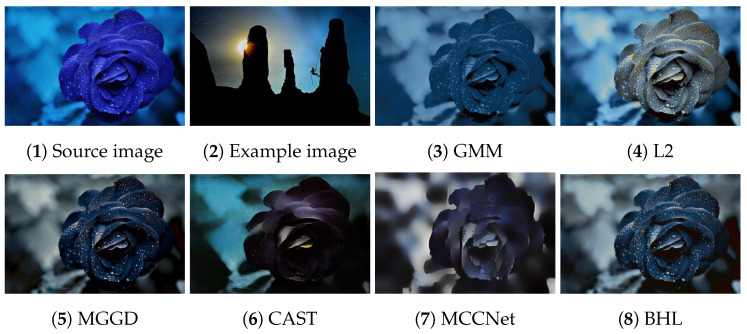
Source image: blue flower; Example image: mountain.

**Figure 4:**
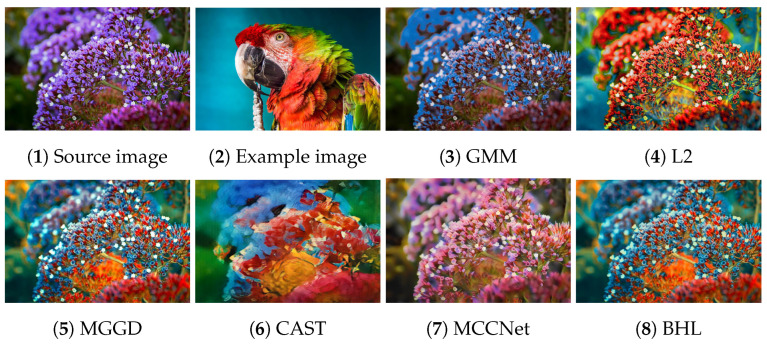
Source image: clusters; Example image: parrot.

**Figure 5:**
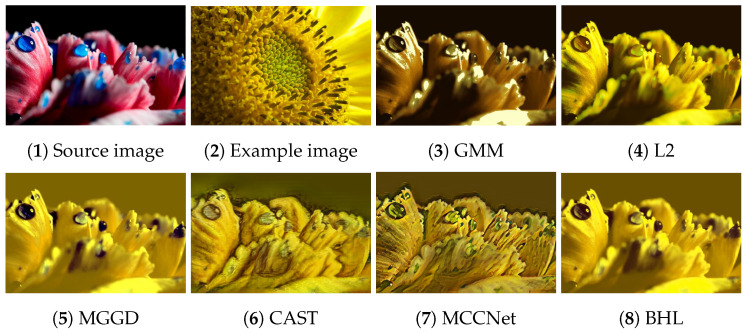
Source image: pink flower; Example image: sunflower.

**Figure 6:**
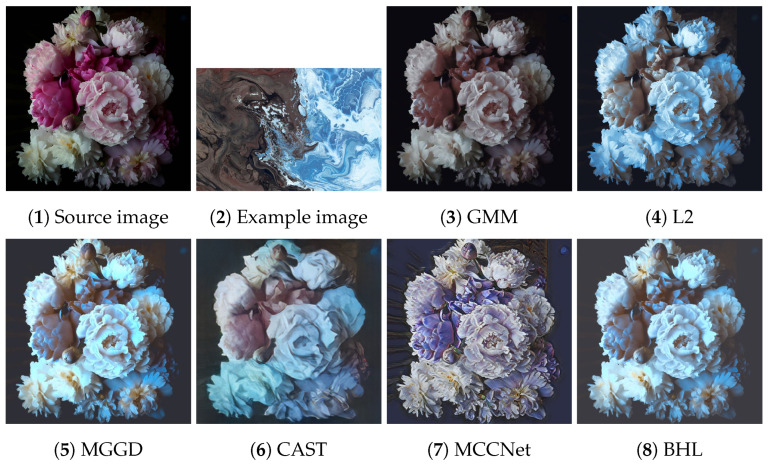
Source image: bouquet; Example image: seaside.

**Figure 7:**
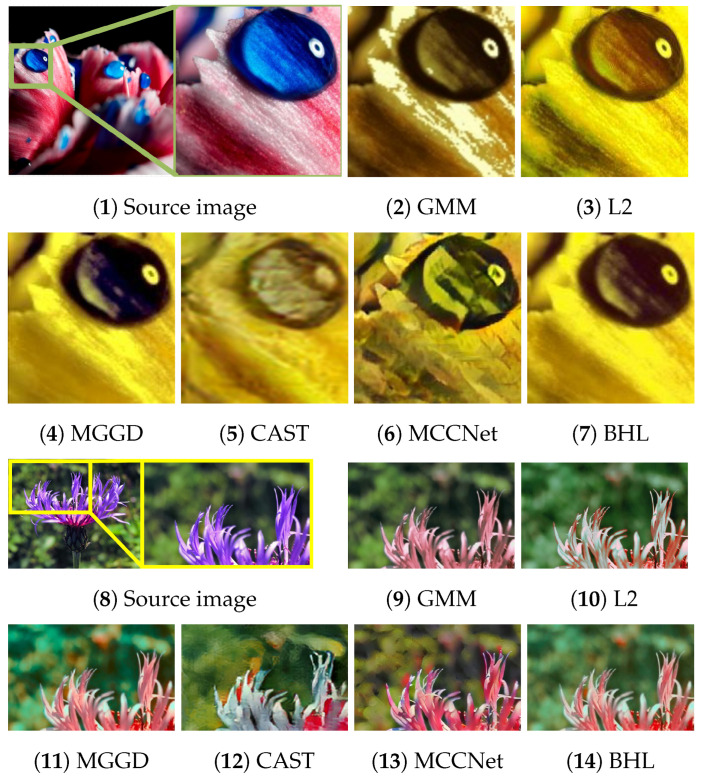
Comparison of details with zoomed-in images.

Figure 2, Figure 3, Figure 4, Figure 5 and Figure 6 present the visual results of the five experiments. Due to its modeling approach, the GMM method excelled at preserving the texture of the source images. This advantage was also reflected in the quantitative evaluation. However, its output was highly sensitive to hyperparameter selection, such as the number of iterations (set to 50 for all five experiments, consistent with [46]). As shown in Figure 4(3) and Figure 6(3), the colors of the example images were not accurately transferred, and the results retained the color style of the source images. Furthermore, in Figure 5(3) and Figure 7(2), significant white artifacts can be observed in the GMM results.

Compared to other methods, the L2 method produced a brighter color style in its results. For example, in Figure 3(4), the L2 result has a significantly brighter color style, but it also deviates from the color style of the example image. This is because the L2 method relies on local matching of the color palettes, and the accuracy of color transfer depends on the precision of this local matching. In Figure 7(3), green shadows are visible on the yellow petals in the L2 result. A similar issue occurs in Figure 7(10), where the petals exhibit a cyan tint.

The MGGD method shares some visual similarities with the BHL method, which also employs a similar modeling technique in its first stage. The key distinction between the MGGD and BHL methods lies in the local refinement in the subsequent steps of the BHL method. Specifically, as shown in Figure 7(4), the MGGD method produces unnatural purple hues on the water droplets, and the black area to the right of the droplets appears opaque and blurry due to the lack of local color adjustments.

Leveraging the powerful ability of convolutional neural networks to extract structural features from images, CAST and MCCNet demonstrated excellent structural fidelity. However, due to the inherent limitations of deep neural networks, such as rigidity in input and output sizes and challenges with generalization, some flaws were present in their results. For instance, in Figure 3(6,7), sharp black blocks appear in the background, which should have been blurred. Similarly, in Figure 7(5,6), the edges of the water droplets in the results of both methods appear as rigid straight lines rather than as curves.

The BHL method achieves a good balance between holistic color style and local structural details. By utilizing a more efficient training method (uRIG) and an innovative sampling strategy, it delivers a more robust color transfer effect. The advantages in terms of time efficiency are demonstrated in the subsequent section.

### 4.4. Time Efficiency

We also assessed the runtime efficiency of the six methods, with the results presented in Table 6. For deep learning methods, the runtime is generally divided into two phases: training and inference. In contrast, probabilistic methods estimate parameters dynamically for each individual dataset or image rather than relying on a pre-trained model. Consequently, their runtime cannot be compared to that of deep learning methods in the same manner, and the results are, therefore, presented differently.

For the four probabilistic methods, the first two rows of Table 6 display the average runtimes across five experiments. This analysis accounts for all processing steps involved in the four comparative methods, including data preprocessing, parameter estimation, and color transformation. The total number of pixels across the five image sets (i.e., the sample size for a single experiment) ranged from 5×1025 to 1.6×1026. The results indicate that due to the BHL method’s use of the uRIG method and a novel sampling strategy based on SLIC, its average runtime across the five experiments was only 4.874 s, demonstrating a significant advantage over the other three probabilistic methods.

In contrast, for the two deep learning methods, CAST and MCCNet, the training times were 18 h and 59 h, respectively, with inference times of 0.011 s and 0.013 s. Compared to the probabilistic methods, CAST and MCCNet exhibited a substantial advantage in inference speed. However, the extended training times and the limited scope of the training datasets clearly constrained their ability to address all scenarios. For instance, in Figure 3(6), large black smudges obscure the petal contours, and in Figure 7(5,6), the water droplet contours are transformed into rigid lines. These issues did not arise with any of the four probabilistic methods.

**Figure 8:**
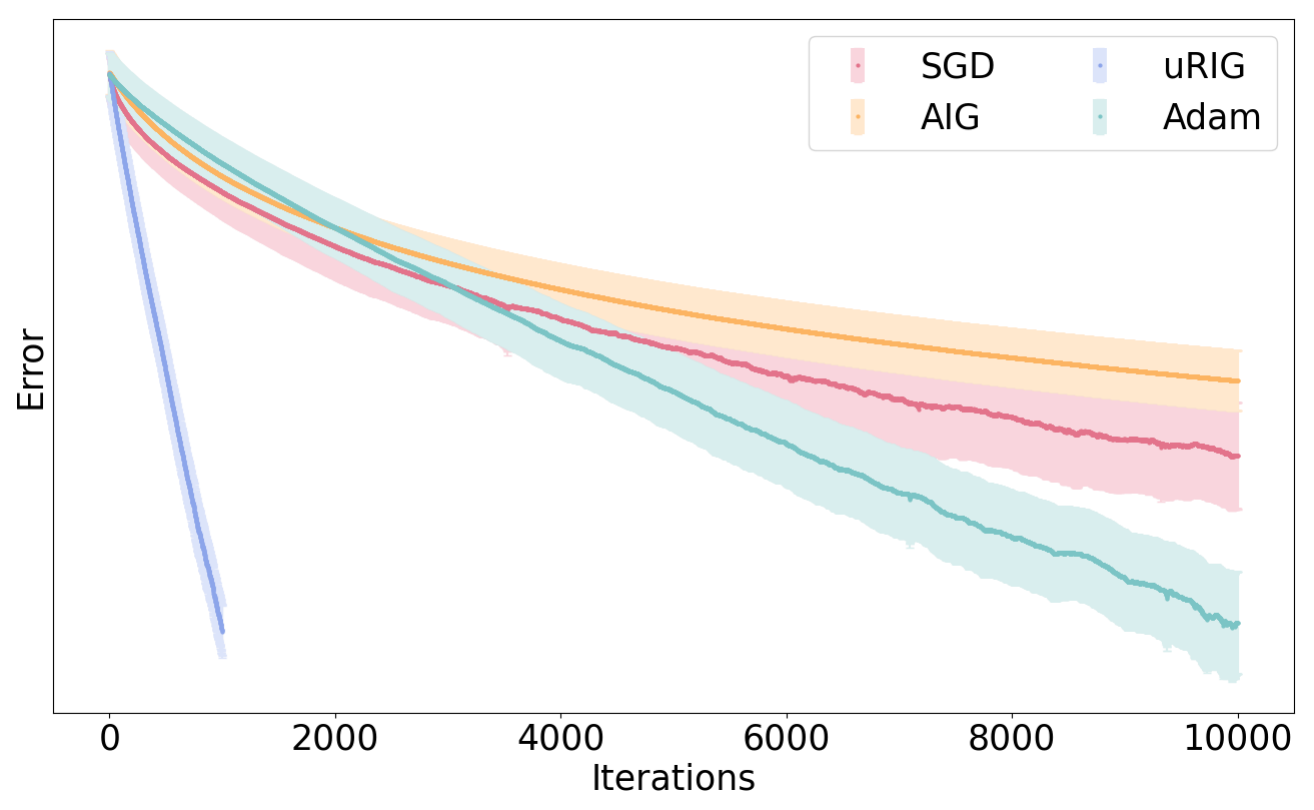
Comparison of uRIG with SGD, AIG, and Adam.

To further validate the time efficiency benefits of the uRIG method, we conducted a simulation comparison experiment with other commonly used stochastic gradient-based optimization methods. The comparison included classic Stochastic Gradient Descent (SGD), Adam [62], and Affine-Invariant Gradient Descent (AIG) [63], which is equivalent to the classic Riemannian gradient descent method. The simulation involved 150 Monte Carlo simulations. Each experiment’s data followed a randomly generated MGGD, with the initial values provided by the method of moments. The averaged results are presented in Figure 8. The horizontal axis represents the number of iterations, while the vertical axis depicts the error calculated using the empirical Kullback–Leibler divergence.

As evident from Figure 8, the uRIG method demonstrates a clear advantage for the parameter estimation task in statistical models. This advantage becomes increasingly pronounced as the number of iterations grows, highlighting the accelerating effect of the Fisher information metric.

## 5. Conclusions

In conclusion, this paper presents a novel color transfer method that effectively balances holistic color style and local detail preservation within a statistical framework. By integrating optimal transport theory in the first stage for holistic color style transfer and utilizing a GMM in the second stage for local detail refinement, the BHL method addresses the inherent challenges of color transfer. The implementation of the unit-wise Riemannian information gradient (uRIG) method successfully tackles the complex optimization problems associated with these stages.

Extensive experimental results demonstrate that the BHL method significantly outperforms existing state-of-the-art techniques in both visual quality and objective evaluation criteria. The proposed method is not only effective but also efficient, with the capability to process high-resolution images in an average time of 4.874 s, making it suitable for practical applications where time constraints are critical.

Overall, the BHL method provides a robust and efficient solution for color transfer in image editing, paving the way for future advancements in the field. Future work may explore further optimization techniques and extend the method to other related image processing tasks.

## Figures and Tables

**Table 1:** X→E:purpleflower→tomato.

Method	GMM [46]	L2 [22]	MGGD [14]	CAST [41]	MCCNet [42]	BHL
SSIM↑	0.972	0.918	0.904	0.610	0.808	0.881
PSNR↑	29.4	22.9	22.1	19.0	22.9	20.3
FID↓	0.037	0.018	0.006	0.014	0.027	0.004
PHV↓	0.730	0.756	0.717	0.691	0.746	0.732
TOPSIS↑	0.260	0.231	0.596	0.211	0.149	**0.809**

The method with the highest TOPSIS score is highlighted in bold. An upward arrow following a criterion indicates that a higher score on this criterion represents better performance. Conversely, a downward arrow indicates that a lower score on this criterion represents better performance.

**Table 2:** X→E:blueflower→mountain.

Method	GMM [46]	L2 [22]	MGGD [14]	CAST [41]	MCCNet [42]	BHL
SSIM↑	0.902	0.782	0.809	0.488	0.574	0.706
PSNR↑	27.6	15.7	24.6	19.5	18.7	22.5
FID↓	0.066	0.119	0.012	0.020	0.020	0.007
PHV↓	0.715	0.758	0.736	0.701	0.682	0.720
TOPSIS↑	0.337	0.187	0.608	0.304	0.308	**0.816**

The bold and arrows are the same as Table 1.

**Table 3:** X→E:clusters→parrot.

Method	GMM [46]	L2 [22]	MGGD [14]	CAST [41]	MCCNet [42]	BHL
SSIM↑	0.957	0.688	0.832	0.477	0.566	0.844
PSNR↑	30.9	16.3	19.5	17.5	16.9	19.9
FID↓	0.056	0.045	0.015	0.016	0.135	0.013
PHV↓	0.792	0.769	0.807	0.733	0.723	0.803
TOPSIS↑	0.475	0.240	0.673	0.525	0.097	**0.707**

The bold and arrows are the same as Table 1.

**Table 4:** X→E:pinkflower→sunflower.

Method	GMM [46]	L2 [22]	MGGD [14]	CAST [41]	MCCNet [42]	BHL
SSIM↑	0.616	0.542	0.404	0.292	0.277	0.434
PSNR↑	22.6	16.3	10.4	11.4	12.6	11.2
FID↓	0.175	0.053	0.007	0.007	0.032	0.003
PHV↓	0.735	0.743	0.684	0.669	0.635	0.662
TOPSIS↑	0.350	0.257	0.317	0.305	0.103	**0.706**

The bold and arrows are the same as Table 1.

**Table 5:** X→E:bouquet→seaside.

Method	GMM [46]	L2 [22]	MGGD [14]	CAST [41]	MCCNet [42]	BHL
SSIM↑	0.775	0.600	0.524	0.318	0.298	0.532
PSNR↑	32.1	17.6	12.9	13.6	14.1	12.5
FID↓	0.186	0.046	0.003	0.012	0.029	0.003
PHV↓	0.712	0.699	0.715	0.675	0.612	0.709
TOPSIS↑	0.457	0.256	0.499	0.156	0.098	**0.589**

The bold and arrows are the same as Table 1.

**Table 6:** Running times of different methods. For the MGGD method, parameter estimation is performed using a fixed-point iteration method.

Probabilistic methods	BHL	L2	GMM	MGGD
Running time	4.874 s	7.523 s	348.754 s	54.897 s
Deep learning methods	CAST		MCCNet	
Training time	18 h		59 h	
Inference time	0.011 s		0.013 s	

## Data Availability

The original contributions presented in the study are included in the article, further inquiries can be directed to the corresponding author.

## Appendix A. Proof of Proposition 1 and Proposition 4

For the Multivariate Generalized Gaussian Distribution (MGGD), the Fisher information metric (FIM) of the scatter matrix Σ for elliptical distributions was first introduced in its general form in [50]. By substituting the parameters specific to the MGGD, the expression in Equation (18b) can be directly obtained. The FIM for the location parameter μ was previously introduced in [51] for the Gaussian distribution. Here, we derive the FIM of μ using the first two derivatives of the log-likelihood function of the MGGD. Recall the log-likelihood function of the MGGD:

(A1) ℓ(x|θ)=lnc−12ln|Σ|+lngβ2(δ)

where the symbol δ and function gβ2 are defined in Equations (5) and (6), respectively. The first and second derivatives of Equation (A1) with respect to μ are

(A2a)dℓ=−2∂lngβ∂δdμ†Σ−1(x−μ)(A2b)d2ℓ=4∂2lngβ∂δ2dμ†Σ−1(x−μ)(x−μ)†Σ−1dμ+∂lngβ∂δdμ†Σ−1dμ

Then, the negative expectation of the second derivative, $-E_{\theta }\left[d^{2}\ell \right]$, provides the FIM with respect to μ. By employing the stochastic expression in Equation (8), a closed-form solution for this integral (expectation) can be obtained:

(A3) $$\begin{array}{cc} -E_{\theta }\left[d^{2}\ell \right] & =-E_{\theta }\left[\frac{4\delta }{d}\frac{\partial^{2}\ln g_{\beta }}{\partial \delta^{2}}+2\frac{\partial \ln g_{\beta }}{\partial \delta }\right]d\mu^{†}\Sigma^{-1}d\mu \\ & =\frac{\left[2(\beta -1)+d\right]\Gamma \left(\frac{d-2}{2\beta }\right)}{2^{\frac{1}{\beta }}d{\left(d-2\right)}^{-1}\Gamma \left(\frac{d}{2\beta }\right)}d\mu^{†}\Sigma^{-1}d\mu \end{array}$$

Finally, for the shape parameter β, which is a positive real number, its Fisher information is also a real-valued scalar. The value of the Fisher information for β is provided in [55] and can be directly used in Equation (18c).

Moving on to Gaussian Mixture Models (GMMs), the FIM for any *m*-th component (m∈{1,⋯,M}) is a special case of Equation (17). By setting μ=zm2, $\Sigma =\sigma_{m}^{2}I$, and β=1, the information coefficient and the FIM with respect to zm2 is

(A4) Izm2uzm2⊤(σm2I)2−1vzm22=uzm2⊤vzm22σm2, Izm=1

For the variance $\sigma_{m}^{2}$, its information coefficients are

(A5) $$I_{\sigma_{m},1}=\frac{1}{2},\;I_{\sigma_{m},2}=0$$

Then, its FIM is

(A6) $$I_{\sigma_{m},1}\mathrm{tr}\left(\frac{u_{\sigma_{m}}v_{\sigma_{m}}}{\sigma_{m}^{4}}I\right)=\frac{du_{\sigma_{m}}v_{\sigma_{m}}}{2\sigma_{m}^{4}}$$

Since the parameter space is a product of these individual units, the uFIM is simply the sum of the FIMs for each unit.

## Appendix B. Proof of Proposition 2 and Proposition 5

The Riemannian information gradient (RIG) of $C_{g}$ with respect to the scatter matrix Σ was established in [56]. The RIG with respect to the location parameter μ associated with uFIM is given by the derivative

(A7) ∇μCh,uμμ=ddεε=0Ch(μ+εuμ)=−uμ†1L∑i=1Lβδiβ−1Σ2−1(xi2−μ)=−1L∑i=1LβIμ−1δiβ−1(xi−μ),uμμ

Solving this equation yields the RIG in Equation (21a). An analogous computation can be applied in the univariate case to obtain the RIG for parameter β.

Each individual component of a GMM is, in fact, a special case of the MGGD, i.e., a Gaussian distribution. Given that $I_{z_{m}}=1$, the RIG with respect to $z_{m}$ is

(A8) $$\begin{array}{cc} \nabla_{z_{m}}C_{\ell }= & -\frac{1}{N}\sum_{n=1}^{N}o_{m,n}I_{z_{m}}^{-1}\delta_{m,n}^{0}\left(e_{n}-z_{m}\right)\\ = & -\frac{1}{N}\sum_{n=1}^{N}o_{m,n}\left(e_{n}-z_{m}\right) \end{array}$$

Then, for $\sigma_{m}$, by solving the following equation:

(A9) $$\frac{d\nabla_{\sigma_{m}}C_{\ell }v_{\sigma_{m}}}{2\sigma_{m}^{2}}=-\frac{1}{N}\sum_{n=1}^{N}\frac{o_{m,n}}{2\sigma_{m}}\left(d-\frac{∥e_{n}-z_{m}{∥}^{2}}{\sigma_{m}}\right)$$

the expression in Equation (25b) can be obtained.

## Appendix C. Proof of Proposition 3 and Proposition 6

In the context of the MGGD, a retraction on the manifold Θ is defined as a smooth mapping Retθ2 from the tangent space Tθ2Θ onto Θ, satisfying the following two specific properties (Definition 4.1.1 of [47]):

1. Retθ2 maps the zero element $0_{\theta }$ of Tθ2Θ back to θ:
  (A10) $$\forall \theta \in \Theta ,\;{\mathrm{Ret}}_{\theta }\left(0\right)=\theta .$$
2. For any element uθ2 in Tθ2Θ, the curve γ(t):t↦Retθ2(tu) satisfies γ˙(0)=uθ2, where
  $$\dot{\gamma }\left(0\right)={\left.\frac{d}{dt}\right|}_{t=0}\gamma \left(t\right).$$
  Setting uθ2=(uμ2,uΣ2,uβ2)2⊤=0θ2, for any point θ, we have
  Retθ(0θ2)=μ+0ΣExpΣ2−10βexp(0/β)=μΣβ=θ
  which verifies property 1. Additionally, for the curve γ(t)=Retθ2(tuθ2),
  γ˙(0θ2)=uμ2ΣExp(Σ2−10Σ2)Σ2−1uΣ2|βexp(0/β)β2−1uβ=(uμ,uΣ,uβ)⊤=uθ2
  which aligns with our choice of t=0. This confirms property 2 for the retraction in Equation (22) of the MGGD.

Similar arguments can be extended to the case of a GMM with multiple components (m=1,…,M). Both properties 1 and 2 can be verified:

(A11a) Retω2(0ω2)=⋮zm2+0zm2σm2exp(0/σm2)⋮=⋮zm2σm2⋮=ω

(A11b) γ˙(0ω2)=⋮uzmσm2exp(0/σm2)σm−1uσm2⋮=⋯,uzm2,uσm2,⋯2⊤=uω2

Since properties 1 and 2 are verified, Equation (26) is a retraction on Ω. Equation (26) is established as a valid retraction in the context of a GMM as well.

## Appendix D. Proof of Proposition 7

In this proof, we utilize the notations introduced in Section 3.4: ω denotes the parameter, and Ω denotes the parameter space. The proof is based on Remark 2 in [56]. Let u(w2(k)) be the current descent direction. According to Remark 2 in [56], if the following condition holds:

(A12c) Eu(w2(k)),∇C(ω2(k))<0, ∀k>0

then, almost surely, we have

(A13) limk→∞ω2(k)=ω*

As we declared in Section 3.4, the cost C(ω) has an isolated stationary point at $\omega =\omega^{*}$, and there exists a compact and convex neighborhood U⊂Ω of $\omega^{*}$ such that the sequence generated by Algorithm 2 (or Algorithm 3) remains within U. For any *k*, we have u(ω2(k))=−∇C(ω2(k)):

(A14) Eu(w2(k)),∇C(ω2(k))=−E∥∇C(ω2(k))∥22<0

This verifies the condition of Remark 2 in [56], and therefore,

(A15) Plimk→∞ω2(k)=ω2*

## References

[1] Kotovenko D., Sanakoyeu A., Lang S., Ommer B. Content and style disentanglement for artistic style transfer. Proceedings of the IEEE/CVF International Conference on Computer Vision.

[2] Li Y., Liu M.Y., Li X., Yang M.H., Kautz J. A closed-form solution to photorealistic image stylization. Proceedings of the European Conference on Computer Vision (ECCV).

[3] Nam S., Ma C., Chai M., Brendel W., Xu N., Kim S.J. End-to-end time-lapse video synthesis from a single outdoor image. Proceedings of the IEEE/CVF Conference on Computer Vision and Pattern Recognition.

[4] Shih Y., Paris S., Durand F., Freeman W.T. (2013). Data-driven hallucination of different times of day from a single outdoor photo. ACM Trans. Graph. (TOG).

[5] Li C., Guo C., Ren W., Cong R., Hou J., Kwong S., Tao D. (2019). An underwater image enhancement benchmark dataset and beyond. IEEE Trans. Image Process..

[6] Li K., Wu L., Qi Q., Liu W., Gao X., Zhou L., Song D. (2022). Beyond single reference for training: Underwater image enhancement via comparative learning. IEEE Trans. Circuits Syst. Video Technol..

[7] Reinhard E., Adhikhmin M., Gooch B., Shirley P. (2001). Color transfer between images. IEEE Comput. Graph. Appl..

[8] Xiao X., Ma L. Color transfer in correlated color space. Proceedings of the 2006 ACM International Conference on Virtual Reality Continuum and Its Applications.

[9] Pitié F., Kokaram A.C., Dahyot R. (2007). Automated colour grading using colour distribution transfer. Comput. Vis. Image Underst..

[10] Pitie F., Kokaram A. The linear Monge-Kantorovitch linear colour mapping for example-based colour transfer. Proceedings of the 4th European Conference on Visual Media Production.

[11] Ferradans S., Papadakis N., Peyré G., Aujol J.F. (2014). Regularized discrete optimal transport. SIAM J. Imaging Sci..

[12] Frigo O., Sabater N., Demoulin V., Hellier P. (2014). Optimal transportation for example-guided color transfer. Proceedings of the Asian Conference on Computer Vision.

[13] Rabin J., Papadakis N. (2015). Non-convex relaxation of optimal transport for color transfer between images. Proceedings of the Geometric Science of Information: Second International Conference (GSI 2015), Palaiseau, France, 28–30 October 2015.

[14] Hristova H., Le Meur O., Cozot R., Bouatouch K. (2017). Transformation of the multivariate generalized Gaussian distribution for image editing. IEEE Trans. Vis. Comput. Graph..

[15] Tai Y.W., Jia J., Tang C.K. Local color transfer via probabilistic segmentation by expectation-maximization. Proceedings of the 2005 IEEE Computer Society Conference on Computer Vision and Pattern Recognition (CVPR’05).

[16] Tai Y.W., Jia J., Tang C.K. (2007). Soft color segmentation and its applications. IEEE Trans. Pattern Anal. Mach. Intell..

[17] Xiang Y., Zou B., Li H. (2009). Selective color transfer with multi-source images. Pattern Recognit. Lett..

[18] Hristova H., Le Meur O., Cozot R., Bouatouch K. Style-aware robust color transfer. Proceedings of the CAe@ Expressive.

[19] Wu F., Dong W., Kong Y., Mei X., Paul J.C., Zhang X. (2013). Content-based colour transfer. Proceedings of the Computer Graphics Forum.

[20] Han Y., Xu C., Baciu G., Li M., Islam M.R. (2016). Cartoon and texture decomposition-based color transfer for fabric images. IEEE Trans. Multimed..

[21] Giraud R., Ta V.T., Papadakis N. Superpixel-based color transfer. Proceedings of the 2017 IEEE International Conference on Image Processing (ICIP).

[22] Grogan M., Dahyot R. (2019). L2 divergence for robust colour transfer. Comput. Vis. Image Underst..

[23] Bonneel N., Sunkavalli K., Paris S., Pfister H. (2013). Example-based video color grading. ACM Trans. Graph..

[24] Su Z., Zeng K., Liu L., Li B., Luo X. (2014). Corruptive artifacts suppression for example-based color transfer. IEEE Trans. Multimed..

[25] Hwang Y., Lee J.Y., So Kweon I., Joo Kim S. Color transfer using probabilistic moving least squares. Proceedings of the IEEE Conference on Computer Vision and Pattern Recognition.

[26] Nguyen R.M., Kim S.J., Brown M.S. (2014). Illuminant aware gamut-based color transfer. Proceedings of the Computer Graphics Forum.

[27] Gong H., Finlayson G.D., Fisher R.B. (2016). Recoding color transfer as a color homography. arXiv.

[28] Gong H., Finlayson G.D., Fisher R.B., Fang F. (2019). 3D color homography model for photo-realistic color transfer re-coding. Vis. Comput..

[29] Wang D., Zou C., Li G., Gao C., Su Z., Tan P. (2017). L0 gradient-preserving color transfer. Proceedings of the Computer Graphics Forum.

[30] Oskarsson M. Robust image-to-image color transfer using optimal inlier maximization. Proceedings of the IEEE/CVF Conference on Computer Vision and Pattern Recognition.

[31] Luan F., Paris S., Shechtman E., Bala K. Deep photo style transfer. Proceedings of the IEEE Conference on Computer Vision and Pattern Recognition.

[32] Liu D., Jiang Y., Pei M., Liu S. (2018). Emotional image color transfer via deep learning. Pattern Recognit. Lett..

[33] Lee J., Son H., Lee G., Lee J., Cho S., Lee S. (2020). Deep color transfer using histogram analogy. Vis. Comput..

[34] Zhou Y., Barnes C., Shechtman E., Amirghodsi S. Transfill: Reference-guided image inpainting by merging multiple color and spatial transformations. Proceedings of the IEEE/CVF Conference on Computer Vision and Pattern Recognition.

[35] Xia X., Zhang M., Xue T., Sun Z., Fang H., Kulis B., Chen J. (2020). Joint bilateral learning for real-time universal photorealistic style transfer. Proceedings of the Computer Vision–ECCV 2020: 16th European Conference, Glasgow, UK, 23–28 August 2020.

[36] Wan D., Shen F., Liu L., Zhu F., Huang L., Yu M., Shen H.T., Shao L. (2020). Deep quantization generative networks. Pattern Recognit..

[37] Liao J., Yao Y., Yuan L., Hua G., Kang S.B. (2017). Visual attribute transfer through deep image analogy. arXiv.

[38] He M., Liao J., Chen D., Yuan L., Sander P.V. (2019). Progressive color transfer with dense semantic correspondences. ACM Trans. Graph. (TOG).

[39] He M., Chen D., Liao J., Sander P.V., Yuan L. (2018). Deep exemplar-based colorization. ACM Trans. Graph. (TOG).

[40] Huang Y., Qiu S., Wang C., Li C. (2020). Learning representations for high-dynamic-range image color transfer in a self-supervised way. IEEE Trans. Multimed..

[41] Zhang Y., Tang F., Dong W., Huang H., Ma C., Lee T.Y., Xu C. Domain enhanced arbitrary image style transfer via contrastive learning. Proceedings of the ACM SIGGRAPH 2022 Conference Proceedings.

[42] Kong X., Deng Y., Tang F., Dong W., Ma C., Chen Y., He Z., Xu C. (2023). Exploring the temporal consistency of arbitrary style transfer: A channelwise perspective. IEEE Trans. Neural Netw. Learn. Syst..

[43] Faridul H.S., Pouli T., Chamaret C., Stauder J., Trémeau A., Reinhard E. (2014). A Survey of Color Mapping and its Applications. Eurographics (State Art Rep.).

[44] Liu S. (2022). An overview of color transfer and style transfer for images and videos. arXiv.

[45] Fang K. (2018). Symmetric Multivariate and Related Distributions.

[46] Gu C., Lu X., Zhang C. (2022). Example-based color transfer with Gaussian mixture modeling. Pattern Recognit..

[47] Absil P.A., Mahony R., Sepulchre R. (2008). Optimization on Matrix Manifolds. Optimization Algorithms on Matrix Manifolds.

[48] Amari S.I. (2016). Information Geometry and Its Applications.

[49] Amari S.I. (1998). Natural gradient works efficiently in learning. Neural Comput..

[50] Berkane M., Oden K., Bentler P.M. (1997). Geodesic estimation in elliptical distributions. J. Multivar. Anal..

[51] Besson O., Abramovich Y.I. (2013). On the Fisher information matrix for multivariate elliptically contoured distributions. IEEE Signal Process. Lett..

[52] Verdoolaege G., De Backer S., Scheunders P. Multiscale colour texture retrieval using the geodesic distance between multivariate generalized Gaussian models. Proceedings of the 2008 15th IEEE International Conference on Image Processing.

[53] Ollivier Y. (2013). Riemannian metrics for neural networks. Inf. Inference J. IMA.

[54] Pennec X., Fillard P., Ayache N. (2006). A Riemannian framework for tensor computing. Int. J. Comput. Vis..

[55] Verdoolaege G., Scheunders P. (2011). Geodesics on the Manifold of Multivariate Generalized Gaussian Distributions with an Application to Multicomponent Texture Discrimination. Int. J. Comput. Vis..

[56] Zhou J., Said S. (2019). Fast, Asymptotically Efficient, Recursive Estimation in a Riemannian Manifold. Entropy.

[57] Burt P.J., Adelson E.H. (1987). The Laplacian pyramid as a compact image code. Readings in Computer Vision.

[58] Wang Z., Bovik A.C., Sheikh H.R., Simoncelli E.P. (2004). Image quality assessment: From error visibility to structural similarity. IEEE Trans. Image Process..

[59] Heusel M., Ramsauer H., Unterthiner T., Nessler B., Hochreiter S. (2017). Gans trained by a two time-scale update rule converge to a local nash equilibrium. Adv. Neural Inf. Process. Syst..

[60] Liu S., Zhang B., Liu Y., Han A., Shi H., Guan T., He Y. (2021). Unpaired stain transfer using pathology-consistent constrained generative adversarial networks. IEEE Trans. Med Imaging.

[61] Alaoui M. (2021). Fuzzy TOPSIS: Logic, Approaches, and Case Studies.

[62] Kingma D.P., Ba J. (2014). Adam: A method for stochastic optimization. arXiv.

[63] Bonnabel S. (2013). Stochastic gradient descent on Riemannian manifolds. IEEE Trans. Autom. Control.

